# A Lens Antenna with Reconfigurable Beams for mmWave Wind Profile Radar

**DOI:** 10.3390/s22093148

**Published:** 2022-04-20

**Authors:** Yafei Ding, Ziwen Zou, Yong Luo, Guangli Yang

**Affiliations:** Shanghai Institute for Advanced Communication and Data Science (SICS), School of Communication and Information Engineering, Shanghai University, Shanghai 200444, China; yafeiding@shu.edu.cn (Y.D.); zwenzou_97@163.com (Z.Z.); y_luo@foxmail.com (Y.L.)

**Keywords:** reconfigurable beams, dielectric lens antenna, wind turbulence, weak signal, high gain, low sidelobe, wind profile radar

## Abstract

Wind profile radar systems require antennas with multiple radiation beams for detecting wind velocity, as well as with a low sidelobe and dual polarization for enhancing the sensitivity for the weak signal reflected from the turbulence. This paper proposes a lens antenna operating at 24 GHz with four reconfigurable beams for wind profile radars. This lens antenna includes 2 × 2 corrugated horn antennas for radiating 24 GHz waves in two polarizations, and the dielectric lens for modulating four radiation beams with a high gain and low sidelobe. Experiments demonstrate that this lens antenna can realize reconfigurable beams with deflections of ±15° in dual polarizations, meanwhile with the gain of 30.58 dBi and the sidelobe of −20 dB. This proposed lens antenna can be applied to mmWave wind profile radars of wind turbines for enhancing wind power efficiency.

## 1. Introduction

In recent years, climate change or global warming has been one of the upcoming focal issues, and green technology for curbing carbon dioxide emissions is in high demand [1]. Wind profile radar utilizing microwave antennas with multiple radiation beams to detect wind turbulence can be one of the green technology candidates applied to wind turbines for wind power [2,3]. Similar technology of wind profiles has been explored for forecasting weather [4,5,6]. In the WPR (Wind Profile Radar), magnitudes and directions of the wind speed are both detected via the antenna with multiple radiation beams pointing to different spatial directions [7,8,9]; thus, DBS (Doppler Beam Steering) antenna [10,11], which requires large-scale phased array antennas, and the Spaced Antenna (SA) antenna [12,13], which resembles MIMO (Multiple-Input Multiple-Output) with setting up several transmitting and receiving antennas, are the typical two types of antenna. In terms of DBS antennas, as shown in [14,15], scanning beams are available but with a mechanical method or multiple antennas. In other references [16,17,18,19], active phase array with electrical beam scanning and operating at around 1280 MHz, 205 MHz, 449 MHz, and 2 GHz have been proposed. In these pioneer works, due to the detected wind turbulence at an altitude as high as 10,000 m, the radar antennas need to work in long wavelengths for mitigating high loss, and low frequency bands around 1 GHz are preferred. Moreover, the WPRs are usually large sizes, up to several or even 10 m; thus, these antennas employing Yagi-Uda and patch design are large and bulky.

However, regarding WPR for altitudes lower than 300 m, which can be applied to wind turbines and wind sites, radars with small sizes are essential; thus, radars with low frequency have the disadvantage of large size, while mmWave can be a good candidate due to its small wavelength, compact size, and high resolution. In [20,21], mmWave phase array antennas operating in the 24–28 GHz band with a compact size have been proposed, but these are for mmWave communications, in which the gains are lower than 30 dBi; otherwise, the array size needs to be very large with plenty of RF chips, resulting in expensive cost and complex manufacturing. Lens antenna is another candidate to realize beam deflection by offsetting focus of the feed as in [22,23,24,25,26,27], which takes the advantages of spherical lens or Luneburg lens to obtain beam steering in 77 or 28 GHz bands. Moreover, a dielectric lens can be shaped to reduce cubic phase errors, thereby realizing low sidelobes, reducing the volume and weight as well [28]. Nevertheless, these lens antennas mentioned above emphasize the antenna properties of beam scanning in large angles, which are suitable for application to short-range detecting for strong signals, while for weak signals such as detecting wind turbulence, the antenna is concerned more on high gains, two polarizations, and low sidelobes.

In this paper, particularly for the WPR for weak signal detecting in the altitude lower than 300 m, a mmWave lens antenna consisting of 2 × 2 corrugated horn antennas operating at 24 GHz, the dielectric lens, and reconfigurable switching control circuit are investigated. Experiments illustrate that the mmWave lens antenna combining dielectric lens and switchable horn array can obtain reconfigurable beams with high gain of 30.58 dBi, low sidelobe of −20 dB, and dual polarizations. It offers a good choice for applying mmWave antenna to the WPR that detects the weak signal of wind turbulence in an altitude lower than 300 m.

## 2. Lens Antenna Design

**Figure 1:**
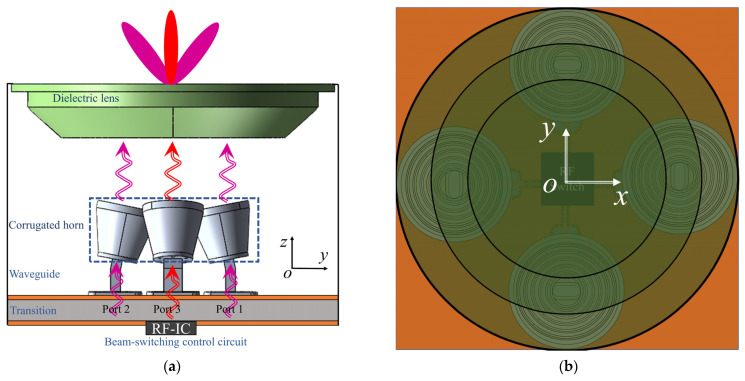
Schematic of the proposed lens antenna with reconfigurable beams. (**a**) Side view. (**b**) Top view.

The proposed lens antenna, as shown in Figure 1, consists of three parts: (1) the radiators providing 24 GHz waves in two polarizations are placed in the middle; (2) the dielectric lens on the top for modulating phases to obtain four reconfigurable beams; and (3) the transformation part with a beam-switching circuit is on the bottom.

### 2.1. Corrugated Horn Antennas

**Figure 2:**
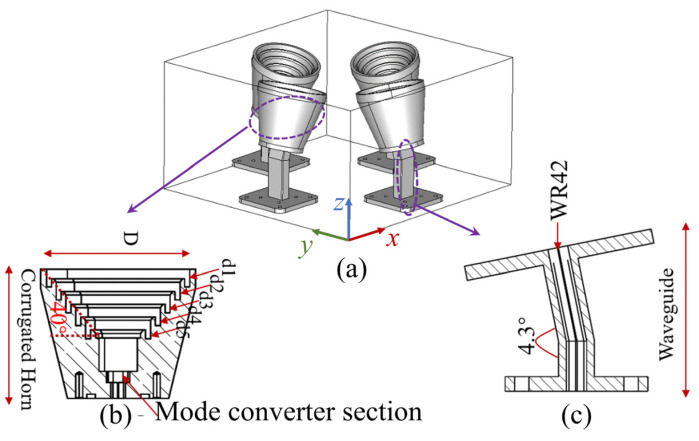
Radiators consists of four horn antennas. (**a**) A 3D review and geometry of the (**b**) corrugated horn and (**c**) waveguide.

**Figure 3:**
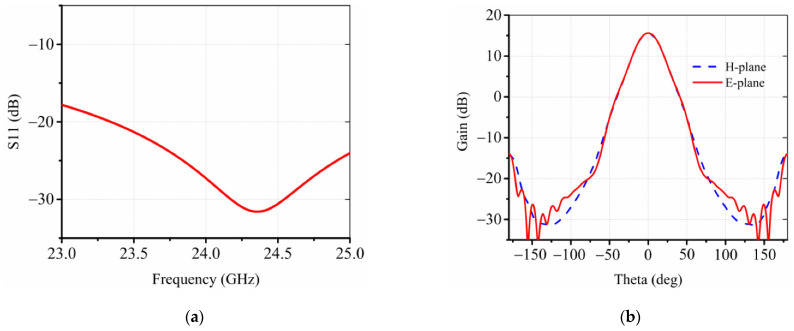
(**a**) Simulated S11 and (**b**) simulated radiation patterns of the proposed horn antenna.

As shown in Figure 2a, the radiator consists of four corrugated horn antennas operating at 24 GHz with 15° tilted angles. Each corrugated horn antenna has five grooves as marked as d1 to d5 in Figure 2b, to generate desired modes of TE11 and TM11, symmetric radiation patterns, and low sidelobes [29]. A WR-42 waveguide propagating the TE10 mode is utilized to realize a smooth connection between the RFIC board and the horn antenna. As shown in Figure 2c, the transition waveguide is designed to have a certain length to eliminate the high order mode caused by the discontinuity of the structure; meanwhile, it has a 4.3° bending angle for the horn antenna to minimize the unwanted phase aberration and improve the projection area of the lens that decreases towards the larger steering angle. More specifically, after the last grooves a short-length mode converter, as shown in Figure 2b, is designed for converting TE10 modes from the waveguide to the TE11 mode in the horn antenna. The corrugations on the side wall of the horn provide the boundary condition to excite the HE11 mode, which is a mixing mode equivalent to the synthesis of TE11 and TM11 modes [29]. The polarization of the corrugated horn is linear polarization, and four horns have rotational symmetry around the center, so that both the vertical and horizontal polarizations can be provided. As shown in Figure 3a, the horn antenna has a wide bandwidth from 23 to 25 GHz, while the gain of radiation patterns is around 15 dBi, as shown in Figure 3b, thereby radiating waves upward to the dielectric lens for modulating the phase and shaping the radiation patterns. Relative parameters in Figure 2 are d1 = d2 = 5.27 mm, d3 = d4 = 5.33 mm, d5 = 5.53 mm, and D = 42.5 mm.

### 2.2. Dielectric Lens

**Figure 4:**
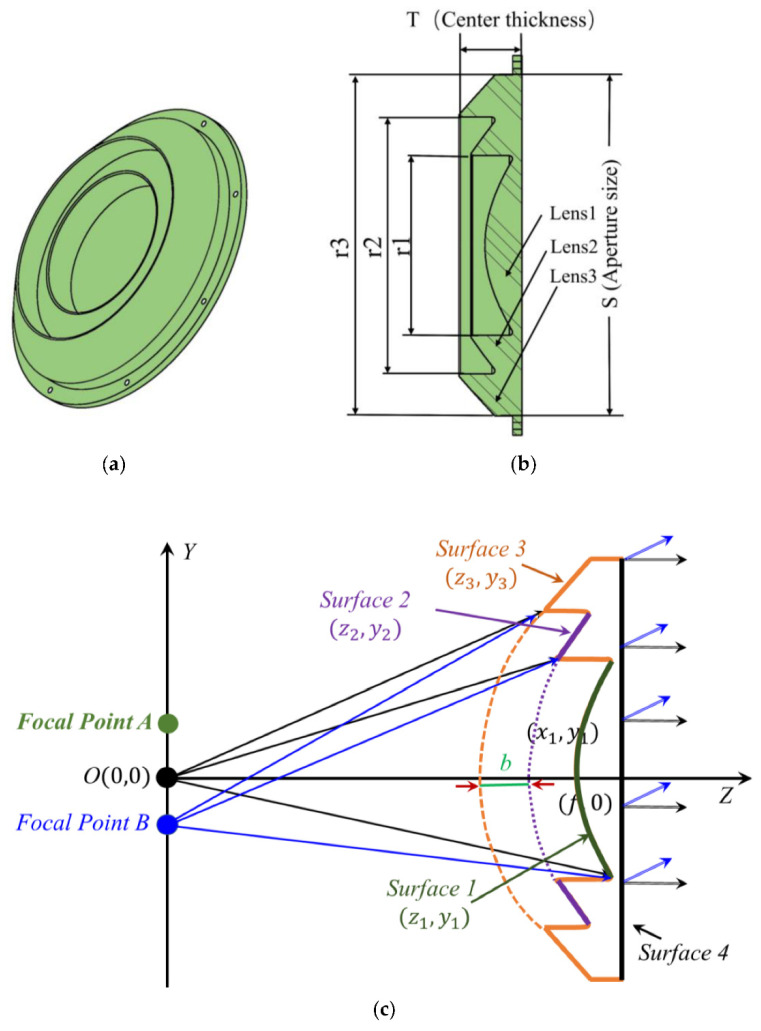
Dielectric lens. (**a**) Simulation structure of the dielectric lens, (**b**) lens geometry, and (**c**) phase path analysis.

As demonstrated in Figure 4a,b, considering the feed source located at O (x = 0, y = 0), the illuminated dielectric lens in the YOZ plane has the focal length f = 200 mm, the center thickness T = 42 mm, and aperture size S = 220 mm. According to the Fermat’s principle, waves travel equal lengths in mediums when they become plane waves, resulting in an inner curve as a hyperbolic curve. The phase distribution will include an inhomogeneous difference such as linear phase difference and cubic phase difference with the feed deviating from the principal axis. Due to the cubic phase error, the sidelobe level of one side of the main lobe is obviously raised. By imposing the Abbe sine condition, the degradation of the sidelobe caused by cubic phase error can be improved. Additionally, according to [30] the Abbe sine condition is fulfilled if the surface of the lens, the one facing the feed, is spherical as shown in Figure 4. The design principle of a coma-correction zoning lens was first explained in [28]. More specifically, usually the lens surface facing the feed is usually spherical, which results in a heavy and bulky dielectric hyperbolic lens and causes the cubic phase error. By designing these grooves and imposing the Abbe sine condition to a dielectric lens antenna, the degradation of the sidelobe caused by cubic phase error can be improved, thereby achieving low sidelobes as well as high gains.

More specifically, as in Figure 4a,b, three spherical surfaces are designed, namely, surface 1, surface 2, and surface 3, which meet the conditions of equal propagating length. Since the wave travels an equal length as it becomes plane waves after propagating through the dielectric lens, these three surfaces 1, 2, and 3 in Figure 4c are described as

(1) $$y_{1}{}^{2}=0.93z_{1}{}^{2}+155.7z_{1}$$

(2) $$y_{2}{}^{2}=0.93{\left(z_{2}+b\right)}^{2}+130.7\left(z_{2}+b\right)$$

(3) $$y_{3}{}^{2}=0.93{\left(z_{3}+2b\right)}^{2}+105.7\left(z_{3}+2b\right)$$

where *b* = λ/(n − 1), so that the degradation of the sidelobe caused by cubic phase error can be improved, meanwhile decreases the volume, thereby reducing the weight. For simplicity, surface 4 is designed to be flat. The dielectric lens is made from Teflon material with permittivity 1.93 and loss tangent 0.0008.

**Figure 5:**
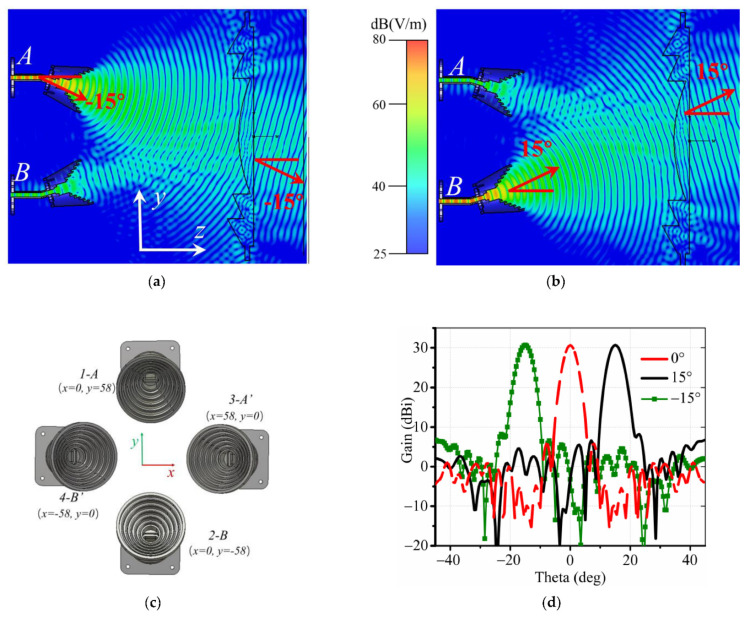
*E*-field distribution of the dielectric lens in the yoz plane at 24 GHz with (**a**) −15 deg, (**b**) 15 deg, (**c**) the 2 × 2 horn array in the xoy plane, and (**d**) radiation patterns.

Furthermore, as shown in Figure 5a, for another focal point A (x = 0, y = 58 mm) that is shifted from the referenced point O (x = 0, y = 0), the wave follows the same propagation path, but the radiation beam tilts to a certain angle −15° to the z-direction as well. Similarly, as demonstrated in Figure 5b, the other horn antenna located downward at B (x = 0, y = −58 mm) produces waves in the 15° direction, and the transmitting waves through the lens go to the 15° direction. Therefore, two antennas in H-polarization and another couple of antennas in V-polarization are employed in the focal zone with different locations A (x = 0, y = 58 mm), A’ (x = 58, y = 0 mm), B (x = 0, y = −58 mm), B’ (x = −58, y = 0 mm), as shown in Figure 5c; thus, we can realize four reconfigurable beams, as shown in Figure 5d. Relative parameters in Figure 4 are r1 = 116 mm, r2 = 165.8 mm, and r3 = 220 mm.

### 2.3. SIW (Substrate Integrated Waveguide)-to-Waveguide Transition

**Figure 6:**
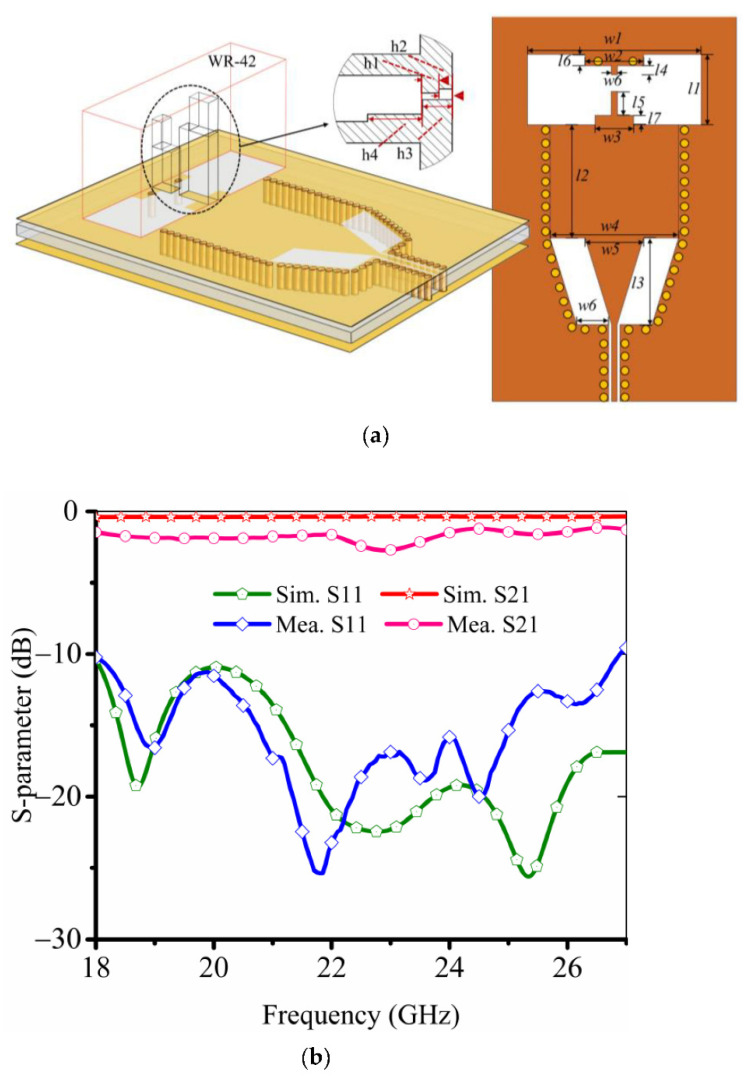
Configuration of the proposed SIW cavity. (**a**) Geometry of the SIW-to-waveguide transition structure. (**b**) Simulated and measured S11, S21 (w1 = 10.668, w2 = 3.6, w3 = 2.36, w4 = 7.87, w5 = 3.59, w6 = 2.14, l1 = 4.318, l2 = 17.4, l3 = 5.3, l4 = 0.57, l5 = 1.47, l6 = 0.7, l7 = 0.58, h1 = 1.64, h2 = 1.24, h3 = 2.8, h4 = 5.13. Unit: mm).

To integrate with the circuit, the SIW-to-waveguide transition is designed to feed the corrugated horn. Generally, the transition for the millimeter wave system is the microstrip-to-waveguide structure [31,32], which requires the extra 1/4 waveguide wavelength of the short-circuit waveguide. However, in the practical application, due to the size limitation, the waveguide needs to be designed to obtain a wideband transition structure at the K-band. As in Figure 6a, the proposed transition structure consists of three parts: SIW cavity, double-ridged waveguide, and grounded coplanar waveguide transmission line (GCPW). The substrate is 0.168-mm-thick Rogers 4350B. The double-ridged waveguide has a large cutoff frequency of main mode (TE_10_) and a wide bandwidth from 18 to 27 GHz, as demonstrated in Figure 6b, covering the frequency bandwidth of the lens antenna. In summary, the whole lens antenna system has good performance from the horn antenna source, dielectric lens, and the transformation part.

## 3. Experimental Demonstration

**Figure 7:**
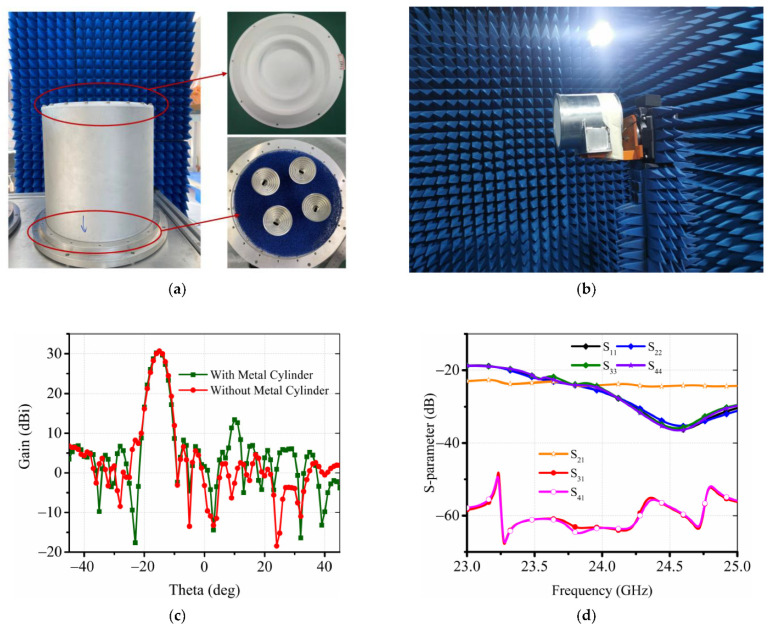
Photograph of the measurement environment (**a**) and the fabricated dielectric lens antenna prototype (**b**). (**c**) Radiation patterns with or without a metal cylinder. (**d**) Simulated S-parameter of the entire dielectric lens antenna.

As shown in Figure 7a, the lens antenna is fabricated as in the model of Figure 1 and measured in the microwave anechoic chamber as in Figure 7b. In case of the −15° beam, the metal cylinder causes the sidelobe level performance to deteriorate as shown in Figure 7c. The antenna is mounted in a metal cylinder with absorbent cotton inside. The 2 × 2 horn array is rotationally symmetric, namely beam 1 and beam 2 (phi = 0°) are vertically polarized and beam 3 and beam 4 (phi = 90°) are horizontally polarized in practice, thereby realizing the four reconfigurable beams in two polarizations at 23, 24, and 25 GHz. The simulated S-parameters in Figure 7d demonstrate that the bandwidth covers the bandwidth of the WPR from 23 to 25 GHz, while the co-polarization isolation between port 1 (port 3) and port 2 (port 4) is lower than −24 dB, and the isolation of cross-polarization between port 1 (port 2) and port 3 (port 4) is lower than −60 dB.

**Figure 8:**
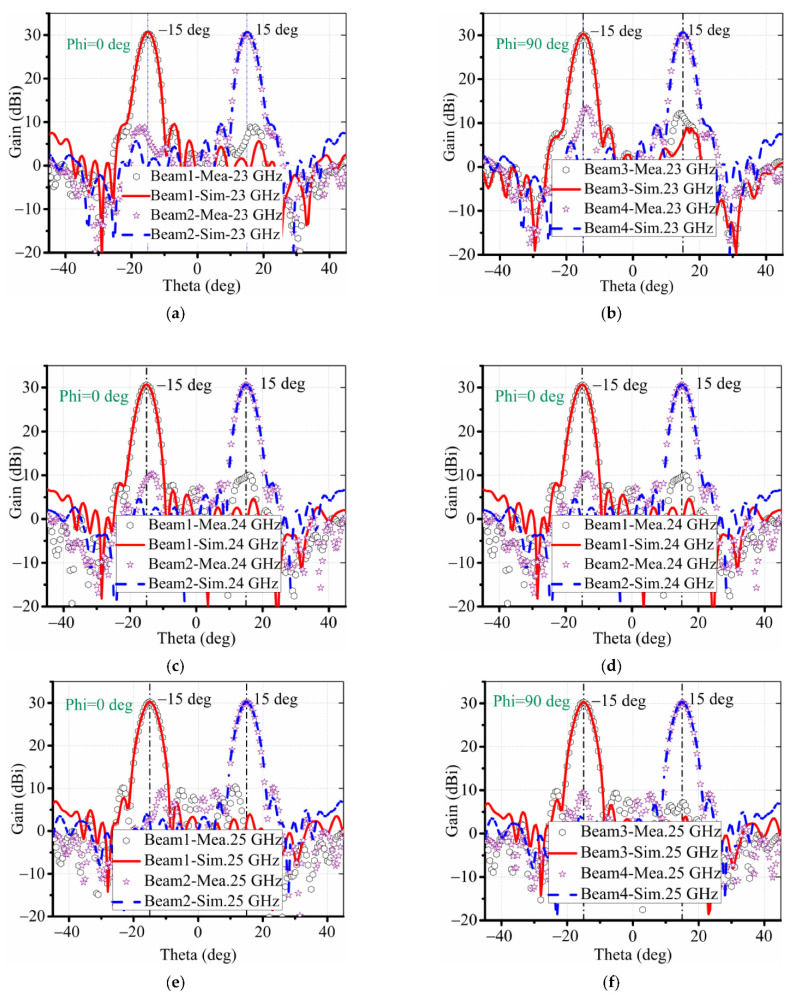
Radiation patterns of different frequencies with different beams. (**a**,**b**) 23 GHz. (**c**,**d**) 24 GHz. (**e**,**f**) 25 GHz.

The measured and simulated radiation patterns are shown in Figure 8, which illustrates a good agreement between simulations and measurements in all four beams. The measured gain and sidelobe from 23 to 25 GHz stabilizes around 30 dB and the sidelobe level is below −20 dB, validating the design with four reconfigurable beams in both H- and V-polarization.

The comparisons among the proposed lens antenna and other relative works are shown in Table 1. It can be found that the proposed antenna is characterized by high gain over 30 dBi, sidelobe lower than −20 dB, two polarizations, while other relative works [22,23,25,27] concern more on radiation beam scanning to large angles, which is suitable for applying to the detection of targets in large range, such as in internet of vehicles, rather than for weak signal detection requiring multiple radiation beams with high gain, and low sidelobe in two polarizations. The proposed mmWave lens antenna can be applied to WPR for detecting weak signals of wind turbulence with heights less than 300 m.

## 4. Conclusions

In the paper, a dielectric lens antenna with four reconfigurable beams was designed. The 2 × 2 horn antenna array was utilized to realize four reconfigurable beams at 24 GHz with two polarizations. The measured sidelobe level is below −20 dB and the realized gain is above 30 dBi from 23 to 25 GHz, demonstrating good agreement with the simulated results. Owing to its multi-beam, dual polarization, high gain, and low sidelobe, the antenna is attractive for WPR detecting weak signals of wind turbulence in low altitudes less than 300 m.

## Figures and Tables

**Table 1:** Comparison with other relative works.

Ref.	Freq. (GHz)	Gain (dBi)	Sidelobe (dB)	Radiation Beams	Suitable Application
[22]	71−76	>13.1	<−12	±40°	strong signal detection
[23]	27.3	19	<−10	±45°/60°	5G
[25]	26	17.4	−15.1	\	communication systems
[27]	77	>30	<−15	±30°	strong signal detection
This work	24	>30	<−20	reconfigurable four beams in ±15°	weak signal detection WPR (height < 300 m)

## Data Availability

The data presented in this study are openly available.
